# Shaping Tomorrow's Surgeons: The Need for Orthopaedic Reform at United Kingdom Medical Schools

**DOI:** 10.7759/cureus.84298

**Published:** 2025-05-17

**Authors:** Daniel I Koshy, David Koshy, Kevin Vinod Joseph, Zabrang Ishaku, Kayaththery Varathan

**Affiliations:** 1 Trauma and Orthopaedics, Royal London Hospital, London, GBR; 2 Orthopaedics, University Hospital Plymouth, Plymouth, GBR; 3 Orthopaedics, St John of God Midland Public and Private Hospitals, Perth, AUS; 4 Internal Medicine, Addenbrooke's Hospital, Cambridge University Hospitals NHS Foundation Trust, Cambridge, GBR

**Keywords:** education, medical curriculum, musculoskeletal medicine, orthopaedic education, trauma and orthopaedics, uk medical schools

## Abstract

Musculoskeletal disorders account for a significant portion of healthcare consultations in the UK, yet orthopaedics remains markedly underrepresented in medical school curricula. This disparity limits students' clinical exposure, practical experience, and research opportunities, contributing to reduced interest in orthopaedic careers and exacerbating workforce shortages. This article explores the current limitations of orthopaedic education, highlighting the consequences for patient care and future workforce planning. It proposes a multifaceted reform strategy that includes simulation-based learning, structured mentorship, curriculum redesign, and national standardisation. Enhancing orthopaedic training is essential to ensure equitable, high-quality musculoskeletal care and to prepare a capable, innovative surgical workforce for the future.

## Introduction and background

Orthopaedics plays a pivotal role in managing musculoskeletal disorders, which account for a significant proportion of healthcare consultations worldwide. The World Health Organization (WHO) estimates that musculoskeletal conditions are the leading contributors to disability worldwide, affecting over 1.7 billion people [1]. In the UK alone, musculoskeletal conditions are responsible for nearly 30% of all general practice consultations, with conditions such as osteoarthritis, back pain, and fractures being among the most common reasons for seeking medical attention [2]. These conditions lead to diminished quality of life and substantial economic burdens, including healthcare costs and lost productivity. Despite the critical importance of orthopaedics, its representation in UK medical school curricula remains insufficient, with limited clinical exposure and inadequate curriculum time dedicated to the speciality [1]. This disconnect poses challenges in attracting students to orthopaedic careers and ensuring a competent workforce to address the growing burden of musculoskeletal conditions. This essay examines the current state of orthopaedic education in UK medical schools, the consequences of its deficiencies, and strategies to enhance exposure and training. Orthopaedics plays a pivotal role in managing musculoskeletal disorders, which account for a significant proportion of healthcare consultations worldwide.

## Review

Current state of orthopaedic education

Orthopaedic education in the UK has evolved significantly over the past century, reflecting broader changes in medical training and healthcare priorities. Historically, musculoskeletal medicine was a prominent component of the medical curriculum, particularly during the early 20th century when infectious diseases such as tuberculosis often manifested in the skeleton. The post-war period saw a surge in orthopaedic advancements, including the development of modern joint replacement surgery and fracture fixation techniques. However, as medical education expanded to incorporate an ever-growing body of knowledge across specialties, orthopaedics began to lose prominence. By the late 20th century, the focus of undergraduate education had shifted toward generalist competencies and public health, relegating orthopaedics to a limited role in the curriculum [3]. Today, orthopaedic education is often integrated into broader surgical or musculoskeletal modules, with considerable variability in the time and resources dedicated to the specialty across medical schools. This historical backdrop underscores the challenges of balancing comprehensive medical training with the need to adequately prepare students for specialities like orthopaedics, which continue to address a substantial burden of disease and disability.

Current challenges in orthopaedic education

Limited Curriculum Time

The average UK medical school allocates minimal time to orthopaedics within clinical rotations. A survey by Al-Nammari et al. [1] found that medical students receive only 1-2 weeks of orthopaedic exposure during their clinical years. This limited exposure contrasts starkly with the prevalence of musculoskeletal conditions in clinical practice, which account for up to 30% of primary care consultations [2]. Although musculoskeletal conditions represent a significant portion of the global disease burden and are a common reason for primary care consultations, orthopaedics often receives limited dedicated time in the undergraduate curriculum [4]. This may be due to the need to balance a wide range of essential disciplines within a constrained timetable, with priority frequently given to areas considered more immediately life-threatening or broadly applicable to generalist medical practice. Consequently, orthopaedic education is sometimes delivered in a more integrated or condensed format, which may limit students’ opportunities for in-depth learning in this field.

Lack of Standardisation

There is significant variability in the content and quality of orthopaedic teaching across UK medical schools. Some institutions incorporate dedicated musculoskeletal modules, while others integrate orthopaedics into broader surgical or general practice rotations, diluting their focus [5]. This inconsistency leaves students with varying levels of exposure and understanding, depending on their medical school.

Insufficient Practical Experience

Hands-on experience is crucial for developing surgical skills and understanding the practical aspects of orthopaedic care. However, many students report limited opportunities to observe or participate in orthopaedic procedures, such as fracture management or joint replacement surgeries [6]. The lack of practical exposure is further compounded by the competition for theatre time among students, trainees, and junior doctors.

Minimal Research Exposure

Orthopaedic research is often underemphasised in the undergraduate curriculum. Many medical students are unaware of the potential for research within the field, and limited opportunities exist for students to engage in musculoskeletal research projects during their studies [7]. This lack of research exposure can impact the development of innovation and evidence-based practice in the specialty [7,8].

Implications of limited orthopaedic exposure

Workforce Shortages

The limited exposure to orthopaedics in medical school contributes to a lack of interest in the specialty, exacerbating workforce shortages. The British Orthopaedic Association (BOA) predicts a shortfall of orthopaedic surgeons in the coming decades, with recruitment failing to keep pace with rising demand [9]. This shortfall is particularly concerning given the aging population and the increasing incidence of conditions such as osteoarthritis and osteoporosis, which require orthopaedic intervention [10].

Suboptimal Patient Care

Inadequate orthopaedic training among general practitioners (GPs) and other non-specialist clinicians can lead to misdiagnosis or delayed referrals. For example, conditions such as slipped capital femoral epiphysis or osteomyelitis may be overlooked due to insufficient musculoskeletal knowledge [11]. Such oversights can delay appropriate treatment, resulting in worse patient outcomes and higher healthcare costs.

Barriers to Research and Innovation

A lack of early exposure to orthopaedics may hinder the development of future clinician-scientists in the field. Research is essential for advancing orthopaedic care, yet many medical students are unaware of research opportunities within the specialty [12]. The lack of exposure to orthopaedic research during medical school may discourage students from pursuing academic careers.

Impacts on Career Choice

Medical students often choose their specialties based on their experiences during clinical placements. Limited exposure to orthopaedics means fewer students develop an interest in the field, contributing to the ongoing recruitment challenges faced by the specialty [13]. This lack of interest is particularly concerning given the highly competitive nature of surgical training programs, where orthopaedics must vie with other specialties for top candidates.

Proposed solutions

Simulation-Based Learning

Simulation-based education offers a safe and effective way to teach orthopaedic skills. Techniques such as virtual reality (VR) surgery and artificial bone models can enhance understanding and procedural competency. These methods provide students with a risk-free environment to practice complex procedures and develop technical skills. Countries like the United States have integrated simulation extensively into medical training. For instance, the University of Pittsburgh Medical Centre reported that simulation-based arthroscopy training reduced error rates among trainees by 40%, underscoring its utility in enhancing surgical proficiency [14]. Similarly, in Singapore, high-fidelity simulators for fracture fixation procedures demonstrated a marked improvement in students' confidence and competence, with 85% of participants rating the experience as highly beneficial [15]. These findings underscore the utility of simulation-based training in enhancing surgical proficiency and clinical competence, supporting its integration into medical education curricula.

Mentorship Programs

Mentorship can raise interest in orthopaedics and guide students to consider a career in the specialty. Structured mentorship programs pairing medical students with orthopaedic surgeons can offer invaluable insights into the field. These programs provide opportunities for students to observe surgeries, discuss career pathways, and gain exposure to orthopaedics. In Canada, the Mentorship in Orthopaedics for Medical Students (MOMS) initiative pairs students with practicing orthopaedic surgeons, significantly increasing exposure to clinical practice and research opportunities [16]. The Australian Orthopaedic Association (AOA) offers a Younger Surgeon Mentoring Program that facilitates mentorship between trainees and experienced orthopaedic surgeons, aiming to support career development and professional growth [17].

The BOA has piloted mentorship initiatives that connect trainees with established surgeons, fostering early engagement and career development [18]. The existence and structure of these mentorship initiatives underscore their importance in guiding students toward orthopaedic surgery.

Curriculum Redesign

Integrating orthopaedics more prominently into the medical curriculum is essential. This could involve dedicated musculoskeletal modules that ensure all students receive comprehensive training in musculoskeletal medicine. A systematic review and meta-analysis highlighted that musculoskeletal education significantly enhances knowledge acquisition and self-confidence among medical students. The study found that students exposed to structured musculoskeletal education demonstrated improved understanding and confidence in managing musculoskeletal conditions compared to those who underwent traditional integrated teaching methods [19].

Extended clinical rotations will provide longer placements in the orthopaedic department. This allows students to gain hands-on experience and a deeper understanding of the specialty. Such immersive experiences can foster greater interest and proficiency in orthopaedics, addressing the current gaps in musculoskeletal education.

Interdisciplinary teaching will aim to collaborate with other teams, such as physiotherapists, rheumatologists and radiologists, to provide a holistic understanding of musculoskeletal care. Integrating pathology and radiology learning into musculoskeletal modules has been shown to enhance students' ability to correlate clinical findings with imaging and pathological data, leading to a more comprehensive clinical education [20].

Increasing Research Opportunities

Promoting research opportunities within medical schools can indeed spark interest in orthopaedics. While specific data from Versus Arthritis indicating that students involved in musculoskeletal research are 60% more likely to consider a career in orthopaedics could not be located, the organisation offers resources aimed at enhancing musculoskeletal education for medical students. For instance, their "Guide to the Clinical Assessment of Patients with Musculoskeletal Conditions" is designed to support medical, nursing, and allied healthcare professional students in developing competencies in musculoskeletal assessment [21].

In the United States, the American Orthopaedic Association's (AOA) Student Scholars Program provides grants for musculoskeletal research projects to medical students. Participants have reported that these experiences solidified their interest in the specialty and improved their academic profiles. Additionally, a study published in the Journal of the American Academy of Orthopaedic Surgeons Global Research & Reviews identified various orthopaedic research fellowship opportunities for medical students, highlighting the benefits of such programs in fostering academic productivity and interest in orthopaedics [22].

Expanding programs like the UK's Academic Foundation Programme (AFP) to include more orthopaedic research opportunities could similarly expose students to cutting-edge research and foster an interest in academic orthopaedics. By integrating research experiences into medical education, institutions can encourage participation and potentially increase the number of students pursuing careers in orthopaedics.

Implications for workforce planning and patient care

Enhancing Workforce Diversity

Enhancing workforce diversity in orthopaedic surgery is crucial, and integrating orthopaedic education more comprehensively into medical curricula can play a significant role. Enhancing workforce diversity in orthopaedic surgery is crucial, and integrating orthopaedic education more comprehensively into medical curricula can play a significant role. By providing targeted outreach programs for underrepresented groups such as women, ethnic minorities and individuals from socioeconomically disadvantaged backgrounds, opportunities can be created for a broader range of students to explore and excel in this specialty.

In the United States, several initiatives have successfully diversified the surgical workforce:

American Academy of Orthopaedic Surgeons (AAOS) IDEA Grant Program: This program supports diversity, equity, and inclusion efforts in orthopaedics by providing financial support to organisations that offer mentorship and scholarship opportunities to underrepresented medical students [23]

Society of Military Orthopaedic Surgeons (SOMOS) E. Anthony Rankin Scholarship Program: This scholarship offers underrepresented medical students the chance to gain exposure to orthopaedic surgery in military settings, providing immersive experiences at institutions like Walter Reed National Military Medical Center [24].

Nth Dimensions Program: Founded by African American orthopaedic surgeons, this program aims to eliminate health disparities by diversifying the physician workforce. It offers internships, symposiums, workshops, and longitudinal mentorship to spark interest in orthopaedics among women and minorities [25].

Replicating such models in the UK could address longstanding disparities and foster a more inclusive orthopaedic community. Implementing structured mentorship programs, providing scholarships, and integrating diversity-focused initiatives into medical education can help achieve this goal.

Addressing Workforce Needs

Enhancing orthopaedic education is pivotal in attracting more medical students to the specialty, thereby addressing workforce shortages. A well-trained orthopaedic workforce is crucial for managing the UK’s aging population and the rising prevalence of musculoskeletal disorders.

The British Orthopaedic Association (BOA) has emphasised the need to increase the number of orthopaedic trainees to meet future healthcare demands. In its Consultant Advisory Book, the BOA notes that despite the increasing demand for musculoskeletal services due to an aging population, the number of trained orthopaedic professionals is decreasing, highlighting a pressing need for more trainees [26]. 

Integrating innovative educational approaches, such as simulation-based learning and interdisciplinary modules, can better prepare medical students for the challenges of modern orthopaedic practice. Simulation training, for instance, has been shown to enhance technical and non-technical skills among orthopaedic trainees, with courses incorporating multidisciplinary scenarios receiving positive feedback for their realism and usefulness [27]. 

By adopting such educational strategies, medical schools can equip future orthopaedic surgeons with the necessary skills and knowledge, ensuring a robust workforce capable of addressing the evolving needs of the UK population.

Improving Patient Outcomes

Certainly, improving musculoskeletal training for general practitioners (GPs) is pivotal in enhancing diagnostic accuracy and early intervention, which can lead to better patient outcomes and reduce the burden on secondary care.

For instance, a Cochrane systematic review titled "Professional interventions for general practitioners on the management of musculoskeletal conditions" examined 30 studies assessing various professional interventions aimed at improving GPs' management of musculoskeletal conditions. The review found that certain interventions, such as GP alerting systems combined with patient-directed education, significantly improved GP behavior regarding diagnostic testing and medication prescribing for osteoporosis. However, the review also noted that the overall quality of evidence was variable, and more research is needed to determine the most effective strategies for other musculoskeletal conditions [28].

Additionally, a study evaluating a GP referral service for manual therapy reported that approximately 65% of patients experienced significant improvement at discharge, and nearly all patients were satisfied with the service. The study also observed a reduction in primary care consultations and inappropriate referrals to secondary care, highlighting the benefits of targeted interventions in musculoskeletal care [29].

These findings underscore the potential benefits of expanding musculoskeletal training initiatives across the UK, which could lead to widespread improvements in patient care and healthcare system efficiency.

Long-Term Benefits for the NHS

Investing in orthopaedic education aligns with the NHS's long-term objectives by enhancing surgical training, reducing errors, and improving patient outcomes. Simulation-based learning, in particular, has demonstrated significant benefits in orthopaedic surgical training.

A systematic review highlighted that simulation training in orthopaedics provides a controlled environment for trainees to develop surgical skills, leading to improved confidence and proficiency. This approach minimises risks to patient safety and reduces the need for operating theatre usage, thereby offering potential cost savings for healthcare systems like the NHS [30].

Furthermore, simulation training has been associated with enhanced trainee performance and a reduction in intra- and postoperative complication rates. These improvements not only benefit patient care but also contribute to more efficient resource allocation within the healthcare system [31].

By integrating innovative educational approaches such as simulation-based learning into orthopaedic training programs, the NHS can better prepare its workforce to meet the challenges of modern healthcare, ultimately leading to improved patient outcomes and long-term cost savings.

Implementing a standardised national orthopaedics curriculum, endorsed by the General Medical Council (GMC), could ensure consistent, high-quality musculoskeletal education across all UK medical schools. This would not only reduce variability in training and guarantee adequate exposure to orthopaedics but also foster greater student interest in the specialty and strengthen foundational knowledge essential for clinical practice.

Challenges and limitations

Implementing advanced educational tools like simulation labs requires substantial investment in infrastructure, equipment, and faculty training. For instance, the initial setup cost for a simulation lab can range between $200,000 to $1.6 million, depending on the complexity of the technology and facilities involved. Additionally, ongoing maintenance, software updates, and staffing contribute to the long-term financial commitment required for such facilities [32].

Curriculum redesign in medical schools often encounters resistance due to various factors. Faculty members may be hesitant to adopt new teaching methods or alter established curricula, citing concerns over increased workload or uncertainty about the efficacy of new approaches. Organisational culture, lack of communication, and insufficient involvement in the change process can further hinder the implementation of curriculum reforms [33].

Ensuring equitable access to enhanced orthopaedic education across all medical institutions, especially those in rural areas, presents unique challenges. Rural medical schools may face limitations in resources, faculty availability, and clinical exposure opportunities, making it difficult to provide comprehensive orthopaedic training. Addressing these disparities is crucial to prevent widening the gap in medical education quality between urban and rural settings.

Time constraints within existing curricula also pose a major barrier. Medical schools operate under tightly packed schedules, and integrating new content such as expanded orthopaedic teaching, simulation-based training, or MDT-focused learning often necessitates either extending course duration or displacing existing material. Without careful planning and consensus among stakeholders, this can lead to competition for time between specialties, contributing to resistance and logistical difficulties during implementation.

## Conclusions

The current lack of orthopaedic exposure in UK medical schools carries significant implications for workforce planning, patient outcomes, and the future of the specialty. To meet these challenges, medical education must adopt innovative strategies such as simulation-based training, structured mentorship, and curriculum redesign. These efforts will not only improve clinical competency and engagement among students but also help address ongoing workforce shortages in orthopaedics. Developing a standardized national orthopaedics curriculum endorsed by the General Medical Council (GMC) could ensure consistent, high-quality musculoskeletal education across all medical schools. This would help reduce variability in training and guarantee that all students receive adequate exposure to orthopaedics. Achieving this vision requires collaboration between medical schools, professional bodies like the British Orthopaedic Association (BOA), and healthcare providers. Joint initiatives and long-term investment are essential to ensure orthopaedic education remains a central priority in preparing tomorrow’s doctors to meet the needs of an evolving healthcare landscape.
